# Cardiotonic Steroids as a Potential Novel Approach for Immunomodulation in Inflammatory Bowel Disease

**DOI:** 10.3390/jcm14124132

**Published:** 2025-06-11

**Authors:** Luiz Henrique Agra Cavalcante-Silva, José Marreiro de Sales-Neto, Mariana Mendonça Soares, Davi Azevedo Ferreira, Anna Beatriz Araujo Medeiros, Sandra Rodrigues-Mascarenhas

**Affiliations:** 1 Medical Sciences and Nursing Complex, Federal University of Alagoas, Arapiraca 57309-005, Brazil; luiz.agra@arapiraca.ufal.br; 2 Biotechnology Center, Federal University of Paraíba, João Pessoa 58051-900, Brazil; engenheiro.sales@gmail.com (J.M.d.S.-N.); marimsoaress@gmail.com (M.M.S.); daviazevedoferreira@ltf.ufpb.br (D.A.F.); annabbeatrizz@gmail.com (A.B.A.M.)

**Keywords:** ouabain, digoxin, anti-inflammatory, neutrophils, T cells

## Abstract

Inflammatory bowel disease (IBD) is a chronic condition that significantly impairs the quality of life of millions of individuals. The pathogenesis of IBD is closely linked to dysbiosis of microbiota and the activation of various inflammatory pathways, which are characterized by elevated levels of activated immune cells, such as neutrophils and lymphocytes. While several therapeutic options, including corticosteroids and biologic agents, are available for the treatment of IBD, their efficacy remains limited. Consequently, the development of novel therapies is essential. In this context, cardiotonic steroids, a class of drugs traditionally known for their effects on the cardiovascular system, have gained attention due to their potential immunomodulatory properties. Thus, this review aims to explore the emerging therapeutic potential of cardiotonic steroids in the treatment of IBD.

## 1. Introduction

Inflammatory bowel disease (IBD) is divided into Crohn’s disease and ulcerative colitis—two distinct, variable, and disabling conditions characterized by chronic inflammation, recurrent flare-ups, and a wide range of debilitating symptoms. IBD can be defined as a non-infectious, chronic, and immune-mediated inflammatory disorder that predominantly targets the digestive tract [1,2]. This condition significantly impairs patients’ quality of life due to the corresponding immune dysregulation and sustained inflammatory dysfunction [3]. Several risk factors may contribute to the development of IBD, although none are definitive predictors [4,5].

Although symptoms of IBD most commonly manifest in individuals during their thirties and forties, the condition is also increasingly diagnosed in older adults [4,5]. The incidence of IBD varies substantially across geographic regions and age groups. According to Caron et al. [6], annual incidence rates range from 23.7 to 39.8 per 100,000 in Oceania, 10.5 to 46.1 per 100,000 individuals in Europe, 7.3 to 30.2 per 100,000 in North America, 1.4 to 1.5 per 100,000 in Asia and the Middle East, and 0.2 to 3.7 per 100,000 in South America. Projections estimate that by 2030, the prevalence of IBD could reach several million individuals in certain countries, including over 3.5 million in the United States, more than 700,000 in the United Kingdom, approximately 815,200 in Germany, and 283,930 in Australia [7].

Currently, there is no single standardized tool for diagnosing IBD [8]. Diagnosis typically requires a comprehensive combination of clinical, radiologic, endoscopic, and histopathologic assessments [9]. Over the past decade, multiple biologic agents and small-molecule therapies have become available to induce and maintain remission in patients with IBD [10]. Aiming to treat IBD, various therapies have been developed based on the modulation of these factors. Most existing treatments often involve immunosuppressant, nonsteroidal, anti-inflammatory, and steroidal drugs. These therapies regulate inflammatory signals, one of the central points of the IBD pathophysiology [10,11,12]. The drug and the treatment vary depending on the severity of the disease. For example, aminosalicylates (e.g., mesalazine and sulfasalazine) are used to manage mild-to-moderate disease [13,14,15]. Topical delivery (e.g., rectal suppositories and enema formulations) are used to treat patients with left-sided disease [16]. Oral formulations can be combined with topical formulations to control disease of the transverse and ascending colon [17]. For severe ulcerative colitis, high-dose oral corticosteroids (e.g., prednisone) are usually prescribed until symptoms stop. For severe and fulminant cases, intravenous corticosteroids are treatment mainstays [17].

In selected patients, severe ulcerative colitis may be refractory to aminosalicylates and corticosteroids [18]. Under these circumstances, janus kinase inhibitors (e.g., tofacitinib), integrin antagonists (e.g., vedolizumab and etrolizumab), cyclosporine, and anti-tumor necrosis factor (TNF) drugs (e.g., infliximab, golimumab, and adalimumab) can be used. However, among them, cyclosporine has more adverse effects [15,19,20,21]. Furthermore, antibiotics should be considered during immunosuppressive drug use [17]. Depending on disease severity and the presence of complications such as strictures or fistulas, surgical intervention may be necessary. Surgical procedures may range from segmental resection to total colectomy [22]. Vieujean et al. [23] fully reviewed the therapeutic options for IBD. Although this therapeutic arsenal is used, it is associated with numerous side effects and limited efficacy, highlighting the need for the development of new therapeutic strategies.

## 2. Immunological Insights into IBD

**Figure 1:**
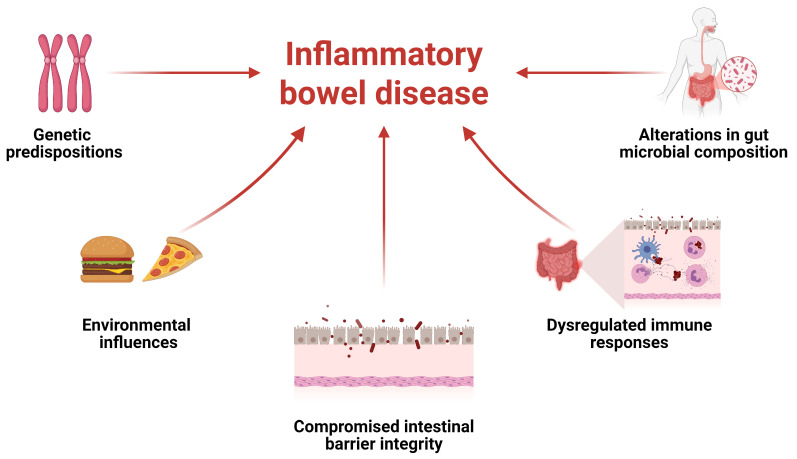
Inflammatory bowel disease (IBD) includes several factors and the interplay between them.

Current evidence suggests that IBD results from a multifactorial interplay between genetic predispositions and environmental influences, ultimately leading to compromised intestinal barrier integrity, dysregulated immune responses, and alterations in gut microbial composition (Figure 1) [24]. The intestinal microbiota serves as a dynamic biological barrier that enhances innate immunity, complementing the mechanical and chemical defenses of the gastrointestinal tract. Disruption of this balance may trigger inflammatory responses and contribute to the onset of various diseases [25]. Although establishing a direct causal relationship between the intestinal microbiota and IBD in humans remains challenging, the interaction between host and microbiota is critical for maintaining immune homeostasis [26].

Aberrant immune responses in IBD are associated with dysregulation of both innate and adaptive immunity [27]. In this regard, innate immune cells such as neutrophils accumulate in inflamed mucosa due to intestinal barrier disruption. They are the most abundant immune cells in human blood and are rapidly recruited to sites of infection or inflammation [27,28]. Their transmigration across the epithelium correlates with disease severity and disruption of crypt architecture in both IBD and experimental colitis [29].

Neutrophils eliminate pathogens through phagocytosis, production of reactive oxygen species (ROS), release of cytotoxic granules (e.g., myeloperoxidase, defensins, lysozyme, neutrophil elastase, proteases, and hydrolases), degranulation, and formation of neutrophil extracellular traps (NETs) [27,30,31]. NET accumulation has been linked to increased TNF-α and interleukin (IL)-1β via the ERK1/2 signaling pathway, exacerbating inflammation in IBD [32]. Neutrophil infiltration also promotes inflammation by generating ROS that damage the epithelial barrier and releasing cytokines such as IL-8, TNF-α, IL-22, and leukotriene B4, which recruit additional neutrophils and monocytes [33,34,35]. Conversely, reducing NET formation can protect against colitis by suppressing pro-inflammatory mediators [32,36]. CD177^+^ neutrophils confer protection in IBD by exerting potent antimicrobial activity while producing lower levels of pro-inflammatory cytokines (e.g., IL-6, IL-17A, IFN-γ) and higher levels of IL-22 and transforming growth factor-β (TGF-β), promoting tissue repair [28,37].

Additionally, in IBD, macrophages exhibit elevated expression of TNF-α, IL-1β, IL-6, IL-12, IL-23, and inducible nitric oxide synthase (iNOS) [38,39,40]. The recruitment of inflammatory C-C chemokine receptor type 2 (CCR2)^+^ monocytes to intestinal tissue contributes to disease severity; notably, CCR2 deficiency ameliorates dextran Sulfate Sodium (DSS)-induced colitis in mice [41]. Moreover, circulating dendritic cells migrate to secondary lymphoid organs and inflamed tissues, where they secrete IL-6, IL-8, and TNF-α, and promote pro-inflammatory T cell phenotypes [42]. Dendritic cell-derived cytokines also modulate epithelial barrier integrity, cell proliferation, and apoptosis via NF-κB2 signaling [43]. In murine DSS-induced colitis, increased mTOR activity correlates with epithelial proliferation in inflamed areas [44,45]. Notably, loss of mTOR signaling in dendritic cells impairs IL-10 production by conventional type 2 dendritic cells, heightening susceptibility to colitis [46,47].

Conventionally, Crohn’s disease is characterized by Th1-mediated immune response, and ulcerative colitis is characterized by Th2-mediated immune response. Furthermore, the Th17-mediated immune response should also be considered, contributing to intestinal inflammation in both forms of IBD, particularly in Crohn’s disease [48,49,50,51,52]. In contrast, Treg cells have tolerizing and anti-inflammatory properties, maintaining self-tolerance, and preventing autoimmunity. Specifically, this maintenance encompasses both a state of tolerance toward self-antigens and the ability to trigger antimicrobial defenses. For this, a few mechanisms act together, including reciprocal regulation of pro-inflammatory effector CD4^+^ T cells and tolerizing anti-inflammatory effects of Treg cells [52,53,54].

Th17 cytokines, such as IL-17A, IL-17F, IL-21, and IL-22, have a dual effect on IBD, attenuating or increasing effects in the gut, as shown in experimental colitis models [55]. For example, adoptive transfer of IL-17A-deficient naïve CD4^+^ T cells to mice with recombination activating gene-1 deficiency results in more severe colitis with higher expression of genes encoding Th1-type cytokines in colon tissue [56]. On the other hand, neutralization of both IL-17A and IL-17F ameliorated colitis at early administration, but administration of IL-17A or IL-17F did not [57]. Nevertheless, secukinumab, a specificIL-17A blocker, exacerbated disease activity in patients with Crohn’s disease [58,59]. Conversely, vidofludimus, an IL-17A and IL-17F blocker, ameliorated both steroid-dependent Crohn’s disease and ulcerative colitis [60]. For a more comprehensive review of IBD immunology, see Uhlig and Powrie [61].

## 3. Cardiotonic Steroids as Potential New Therapeutics in IBD Treatment

As described earlier, the pathogenesis of IBD involves multiple factors. One of them is gut microbiota dysbiosis, which is marked by an increase in harmful bacteria and a reduction in beneficial probiotics. Another factor is the exacerbation of inflammation, facilitated by excessive secretion of pro-inflammatory cytokines, including TNF-α and IL-1β. In addition, there is an increase in intestinal levels of reactive oxygen species, including hydrogen peroxide (H_2_O_2_), hydroxyl radicals (•OH), and superoxide anions (•O^2−^)) [62,63,64]. The pleiotropic mechanisms associated with this disease open up opportunities for the development of multiple targeted therapeutic strategies.

Given the ongoing search for new therapies to treat chronic diseases with limited treatment options, drug repurposing has emerged as a promising strategy. This approach can reduce development time, lower costs, increase clinical success rates, and enable the exploration of novel therapeutic pathways [2]. In this context, cardiotonic steroids have gained attention. They are known to inhibit Na^+^/K^+^-ATPase and to promote a positive inotropic effect [65]. Although these steroids were initially used to treat heart failure and arrhythmias [66], several biological activities have been reported, including anti-inflammatory activity [67,68,69,70]. For that, cardiotonic steroid modulates the immune system mainly by affecting signal transduction [69,71].

Digoxin, an archetypal cardiotonic steroid, is still used for treatment of patients with atrial fibrillation and heart failure in low doses, as reviewed by Fender et al. [72] and Whayne [73]. It was shown that this molecule is used to suppress Th17 cell differentiation by antagonizing RORγt activity [74,75]. Moreover, digoxin also reduced the severity of experimental autoimmune encephalomyelitis in mice [74,75] and experimental arthritis in mice [76]. More recently, it has been shown that digoxin suppresses Th17-related cytokines (IL-17A and IL-17F) in colonic mucosa in murine T cell transfer-induced colitis [77]. Additionally, modulating T cell proliferation may represent a potential strategy for attenuating T cell-mediated pathogenesis. Notably, ouabain—a cardiotonic steroid previously used clinically for the treatment of heart failure—has been shown to influence T cell proliferation, possibly through the downregulation IL-2 production [78].

Ouabain has also been reported to modulate neutrophil function. Studies have shown that this molecule reduces neutrophil migration both in vitro and in vivo in response to various inflammatory stimuli, including zymosan, ovalbumin, and *Leishmania* spp. [67,68,69,79]. The mechanisms underlying this inhibition appear to involve a reduction in CD18 integrin expression and inhibition of the p38 mitogen-activated protein kinase protein. In addition, marinobufagenin, a cardiotonic bufadienolide steroid, also inhibits neutrophil migration into the peritoneal cavity [70]. Given the well-established role of neutrophils in IBD pathogenesis, therapeutic strategies aimed at modulating their activity may represent a promising avenue for treatment [80].

Beyond their effects on cell migration, cardiotonic steroids can also reduce levels of pro-inflammatory cytokines, such as TNF and IL-1β, many of which are associated with IBD [81]. Furthermore, recent studies have shown that a synthetic cardiotonic steroid, γ-benzylidene digoxin 8, upregulates the production of IL-10, an anti-inflammatory cytokine, in peritoneal macrophages [82]. The regulation of various cytokines is linked to the NF-κB and mitogen-activated protein kinase signaling pathways, both of which have been shown to be modulated by cardiotonic steroids. Ouabain has also been shown to reduce vascular permeability induced by inflammatory stimuli [67]. In addition, ouabain modulates tight junctions and gap junctional communication in epithelial cells [83,84]. Based on these findings, it is conceivable that similar mechanisms might contribute to the stabilization of the intestinal epithelial barrier. Although direct evidence in the context of gut epithelium remains limited, this hypothesis warrants further investigation, particularly in the setting of IBD, where epithelial dysfunction plays a central role. Notably, several immunomodulatory effects of cardiotonic steroids have been described in the peritoneal cavity [67,70,79], and, although direct evidence remains to be fully established, this may suggest a potential anti-inflammatory action in intestinal inflammatory conditions, warranting further investigation.

It is noteworthy that the anti-inflammatory and immunomodulatory effects of cardiotonic steroids, particularly ouabain, are not limited to peritoneal inflammation. Ouabain has demonstrated significant anti-inflammatory activity within the central nervous system, notably in the rat hippocampus, where it reduced messenger ribonucleic acid expression levels of iNOS and IL-1β [85] and has also been shown to modulate retinal neuroinflammation [86]. In addition, ouabain exerts protective effects in pulmonary inflammation by reducing cytokine production, inhibiting leukocyte migration, and attenuating tissue remodeling in the lungs [68,87]. Collectively, these findings reinforce the anti-inflammatory potential of cardiotonic steroids and highlight their possible application in the treatment of other inflammatory conditions.

A potential concern regarding the use of cardiotonic steroids is the possibility of dose-dependent side effects. While this is a valid point, it is important to note that many of the immunomodulatory effects of these compounds have been observed at very low doses and concentrations—often insufficient to inhibit Na^+^/K^+^-ATPase, which is primarily responsible for the more severe adverse effects [88]. Moreover, the application of advanced technologies in the development of novel pharmaceutical formulations can help mitigate adverse effects and enhance therapeutic efficacy [89].

As highlighted in a recent review by Snelson et al. [90], disruption of the heart–gut axis contributes to systemic inflammation and is closely linked to the pathophysiology of heart failure. This dynamic connection indicates that chronic inflammatory diseases of the gut, such as IBD, may exacerbate cardiac dysfunction. In this context, the knowledge about the immunomodulatory properties of cardiac glycosides, such as ouabain [67,68,69,70], could offer a therapeutic advantage beyond their classical cardiovascular effects. While these agents are not currently used in the management of IBD, their ability to modulate inflammatory pathways may provide not only local but also systemic benefits, potentially attenuating inflammatory drivers of heart–gut axis dysfunction.

## 4. The Gut Microbiota Plays an Important Role in Cardiotonic Steroid Bioavailability

IBD development has been linked to inflammation induced by certain intestinal microbiota, such as *Clostridium difficile* [91]; species from *Clostridium* cluster XIVa, IV, and XVIII [92,93]; colibactin-producing *Escherichia coli*; and *Fusobacterium nucleatum* [54]. Furthermore, the microbiota also influences the bioavailability of oral drugs, including digoxin [94]. Digoxin can be converted into cardioinactive forms—digoxin reduction products (DRPs)—such as dihydrodigoxin, in which the single double bond in the lactone ring of digoxin is reduced [95,96]. DRP has low affinity for the Na^+^/K^+^-ATPase. Moreover, cardiac tissue has low rate of DRPs, and DRPs can be rapidly excreted, resulting in significantly lower cardiotonic therapeutic efficacy compared to digoxin [95]. Since 1981, when the effect of gut microbiota on digoxin inactivation was first seen [96], several bacteria have been associated with this phenomenon, including *Eggerthella lenta* [95]. The inactivation of digoxin by *E. lenta* was discovered in 2013 [97]. In this context, two proteins were predicted using sequence homology and secondary structure predictions. The first, cardiac glycoside reductase (CGR) 1, exhibits homology with the NapC/NirT family of cytochrome *c* reductases. The second, CGR2, shares structural similarity with fumarate reductases, suggesting a potential role in redox reactions linked to digoxin metabolism [98]. The function of CGR1 is to transfer electrons from quinones to its associated terminal electron reductase partner. For this, CGR1, anchoring CGR2 to the membrane, forms a complex with CGR2, facilitating electron transfer through a flavin adenine dinucleotide redox-dependent mechanism. The digoxin-binding site is present in the CGR2 protein and contains negatively charged polar amino acids and non-polar hydrophobic residues [99]. Some molecules, such as arginine, can inhibit the conversion of digoxin to inactive forms [95,97], required for the growth of *E. lenta* [100]. Therefore, a high-arginine diet may be beneficial, improving digoxin bioavailability [101]. In addition, the gut microbiota can also activate Th17 cells, which can be inhibited by digoxin. For example, human gut *E. lenta* induces intestinal Th17 activation by lifting inhibition of the Th17 transcription factor RORγt. Although Th17 activation varies across *E. lenta* strains, the activation is attributable to CGR2 that induces IL-17A [102].

## 5. Final Considerations and Future Directions

**Figure 2:**
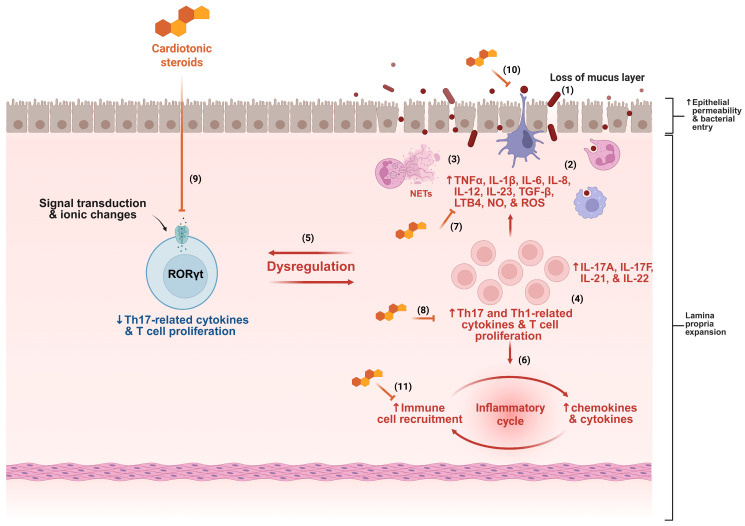
Several mechanisms contribute to the pathogenesis of inflammatory bowel disease (IBD), including disruption of the mucus layer, dysbiosis, and increased epithelial permeability, facilitating bacterial translocation (1). This process triggers immune system activation, characterized by intense cellular infiltration, particularly of neutrophils and monocytes (2), accompanied by the release of various inflammatory mediators and neutrophil extracellular traps (NETs) (3). The presence of T cells with Th1 and Th17 profiles is a hallmark of IBD (4), contributing to the dysregulation of gut homeostasis (5). The inflammatory cycle is perpetuated by continuous cell migration and sustained release of cytokines and chemokines (6). Based on the mechanisms described in the literature, cardiotonic steroids may interfere at multiple points in this inflammatory cascade, including by reducing cytokine production (7), modulating T cell activity (8), dampening Th17 responses (9), preserving epithelial barrier integrity (10), and inhibiting immune cell migration (11). ↑ indicates increase, ↓ indicates decrease, and ⊣ indicates inhibition.

Considering the multiple pathways involved in the pathogenesis of IBD and the pressing need for new therapies to address this complex inflammatory condition (Figure 2), the investigation of cardiotonic steroids, a group of molecules with pleiotropic immunomodulatory effects, emerges as a plausible and promising strategy in the search for novel IBD treatments.

Thus, to advance this approach with a focus on drug repurposing, several strategies can be considered. These include the analysis of electronic medical records and public databases to identify retrospective evidence supporting the efficacy of cardiotonic steroids in IBD; the assessment of the cost-effectiveness of this potential treatment—particularly relevant given that drugs such as digoxin are already in therapeutic use and may offer a financially viable option; and the careful selection of molecular targets and disease biomarkers to evaluate and monitor therapeutic responses.

We also emphasize that the use of cardiotonic steroids in the treatment of IBD remains a hypothesis-generating consideration, and further preclinical and clinical studies are necessary to determine their potential translational applicability in this context.

## References

[1] Farrell D., Artom M., Czuber-Dochan W., Jelsness-Jorgensen L.P., Norton C., Savage E. (2020). Interventions for fatigue in inflammatory bowel disease. Cochrane Database Syst. Rev..

[2] Pushpakom S., Iorio F., Eyers P.A., Escott K.J., Hopper S., Wells A., Doig A., Guilliams T., Latimer J., McNamee C. (2019). Drug repurposing: Progress, challenges and recommendations. Nat. Rev. Drug Discov..

[3] Diez-Martin E., Hernandez-Suarez L., Munoz-Villafranca C., Martin-Souto L., Astigarraga E., Ramirez-Garcia A., Barreda-Gomez G. (2024). Inflammatory bowel disease: A comprehensive analysis of molecular bases, predictive biomarkers, diagnostic methods, and therapeutic options. Int. J. Mol. Sci..

[4] Roda G., Chien Ng S., Kotze P.G., Argollo M., Panaccione R., Spinelli A., Kaser A., Peyrin-Biroulet L., Danese S. (2020). Crohn’s disease. Nat. Rev. Dis. Primers.

[5] Kobayashi T., Siegmund B., Le Berre C., Wei S.C., Ferrante M., Shen B., Bernstein C.N., Danese S., Peyrin-Biroulet L., Hibi T. (2020). Ulcerative colitis. Nat. Rev. Dis. Primers.

[6] Caron B., Honap S., Peyrin-Biroulet L. (2024). Epidemiology of inflammatory bowel disease across the ages in the era of advanced therapies. J. Crohns Colitis.

[7] Kaplan G.G., Windsor J.W. (2021). The four epidemiological stages in the global evolution of inflammatory bowel disease. Nat. Rev. Gastroenterol. Hepatol..

[8] M’Koma A.E. (2022). Inflammatory bowel disease: Clinical diagnosis and surgical treatment-overview. Medicina.

[9] Van Assche G., Dignass A., Bokemeyer B., Danese S., Gionchetti P., Moser G., Beaugerie L., Gomollon F., Hauser W., Herrlinger K. (2013). Second European evidence-based consensus on the diagnosis and management of ulcerative colitis part 3: Special situations. J. Crohns Colitis.

[10] Rogler G., Singh A., Kavanaugh A., Rubin D.T. (2021). Extraintestinal manifestations of inflammatory bowel disease: Current concepts, treatment, and implications for disease management. Gastroenterology.

[11] Chang J.T. (2020). Pathophysiology of inflammatory bowel diseases. N. Engl. J. Med..

[12] Shah S.C., Itzkowitz S.H. (2022). Colorectal cancer in inflammatory bowel disease: Mechanisms and management. Gastroenterology.

[13] Marshall J.K., Thabane M., Steinhart A.H., Newman J.R., Anand A., Irvine E.J. (2010). Rectal 5-aminosalicylic acid for induction of remission in ulcerative colitis. Cochrane Database Syst. Rev..

[14] Ford A.C., Achkar J.P., Khan K.J., Kane S.V., Talley N.J., Marshall J.K., Moayyedi P. (2011). Efficacy of 5-aminosalicylates in ulcerative colitis: Systematic review and meta-analysis. Am. J. Gastroenterol..

[15] Travis S.P., Stange E.F., Lemann M., Oresland T., Bemelman W.A., Chowers Y., Colombel J.F., D’Haens G., Ghosh S., Marteau P. (2008). European evidence-based Consensus on the management of ulcerative colitis: Current management. J. Crohns Colitis.

[16] Cohen R.D., Woseth D.M., Thisted R.A., Hanauer S.B. (2000). A meta-analysis and overview of the literature on treatment options for left-sided ulcerative colitis and ulcerative proctitis. Am. J. Gastroenterol..

[17] Sairenji T., Collins K.L., Evans D.V. (2017). An update on inflammatory bowel disease. Prim. Care.

[18] Faubion W.A., Loftus E.V., Harmsen W.S., Zinsmeister A.R., Sandborn W.J. (2001). The natural history of corticosteroid therapy for inflammatory bowel disease: A population-based study. Gastroenterology.

[19] Akiho H., Yokoyama A., Abe S., Nakazono Y., Murakami M., Otsuka Y., Fukawa K., Esaki M., Niina Y., Ogino H. (2015). Promising biological therapies for ulcerative colitis: A review of the literature. World J. Gastrointest. Pathophysiol..

[20] Ford A.C., Sandborn W.J., Khan K.J., Hanauer S.B., Talley N.J., Moayyedi P. (2011). Efficacy of biological therapies in inflammatory bowel disease: Systematic review and meta-analysis. Am. J. Gastroenterol..

[21] Clark M., Colombel J.F., Feagan B.C., Fedorak R.N., Hanauer S.B., Kamm M.A., Mayer L., Regueiro C., Rutgeerts P., Sandborn W.J. (2007). American gastroenterological association consensus development conference on the use of biologics in the treatment of inflammatory bowel disease, June 21–23, 2006. Gastroenterology.

[22] Axelrad J.E., Lichtiger S., Yajnik V. (2016). Inflammatory bowel disease and cancer: The role of inflammation, immunosuppression, and cancer treatment. World J. Gastroenterol..

[23] Vieujean S., Jairath V., Peyrin-Biroulet L., Dubinsky M., Iacucci M., Magro F., Danese S. (2025). Understanding the therapeutic toolkit for inflammatory bowel disease. Nat. Rev. Gastroenterol. Hepatol..

[24] Bruner L.P., White A.M., Proksell S. (2023). Inflammatory bowel disease. Prim. Care.

[25] Zheng D., Liwinski T., Elinav E. (2020). Interaction between microbiota and immunity in health and disease. Cell Res..

[26] Haneishi Y., Furuya Y., Hasegawa M., Picarelli A., Rossi M., Miyamoto J. (2023). Inflammatory bowel diseases and gut microbiota. Int. J. Mol. Sci..

[27] Saez A., Herrero-Fernandez B., Gomez-Bris R., Sanchez-Martinez H., Gonzalez-Granado J.M. (2023). Pathophysiology of inflammatory bowel disease: Innate immune system. Int. J. Mol. Sci..

[28] Zhou G., Yu L., Fang L., Yang W., Yu T., Miao Y., Chen M., Wu K., Chen F., Cong Y. (2018). CD177^+^ neutrophils as functionally activated neutrophils negatively regulate IBD. Gut.

[29] Kang L., Fang X., Song Y.H., He Z.X., Wang Z.J., Wang S.L., Li Z.S., Bai Y. (2022). Neutrophil-epithelial crosstalk during intestinal inflammation. Cell. Mol. Gastroenterol. Hepatol..

[30] Drury B., Hardisty G., Gray R.D., Ho G.T. (2021). Neutrophil extracellular traps in inflammatory bowel disease: Pathogenic mechanisms and clinical translation. Cell. Mol. Gastroenterol. Hepatol..

[31] Chen H., Wu X., Xu C., Lin J., Liu Z. (2021). Dichotomous roles of neutrophils in modulating pathogenic and repair processes of inflammatory bowel diseases. Precis. Clin. Med..

[32] Maronek M., Gromova B., Liptak R., Konecna B., Pastorek M., Cechova B., Harsanyova M., Budis J., Smolak D., Radvanszky J. (2021). Extracellular DNA correlates with intestinal inflammation in chemically induced colitis in mice. Cells.

[33] Zhou G.X., Liu Z.J. (2017). Potential roles of neutrophils in regulating intestinal mucosal inflammation of inflammatory bowel disease. J. Dig. Dis..

[34] Pavlidis P., Tsakmaki A., Pantazi E., Li K., Cozzetto D., Digby-Bell J., Yang F., Lo J.W., Alberts E., Sa A.C.C. (2022). Interleukin-22 regulates neutrophil recruitment in ulcerative colitis and is associated with resistance to ustekinumab therapy. Nat. Commun..

[35] Biasi F., Leonarduzzi G., Oteiza P.I., Poli G. (2013). Inflammatory bowel disease: Mechanisms, redox considerations, and therapeutic targets. Antioxid. Redox Signal..

[36] Hansberry D.R., Shah K., Agarwal P., Agarwal N. (2017). Fecal myeloperoxidase as a biomarker for inflammatory bowel disease. Cureus.

[37] Seo D.H., Che X., Kim S., Kim D.H., Ma H.W., Kim J.H., Kim T.I., Kim W.H., Kim S.W., Cheon J.H. (2021). Triggering receptor expressed on myeloid cells-1 agonist regulates intestinal inflammation via Cd177^+^ neutrophils. Front. Immunol..

[38] Bain C.C., Schridde A. (2018). Origin, differentiation, and function of intestinal macrophages. Front. Immunol..

[39] Sun R., Abraham C. (2020). IL23 promotes antimicrobial pathways in human macrophages, which are reduced with the IBD-protective IL23R R381Q variant. Cell. Mol. Gastroenterol. Hepatol..

[40] Soufli I., Toumi R., Rafa H., Touil-Boukoffa C. (2016). Overview of cytokines and nitric oxide involvement in immuno-pathogenesis of inflammatory bowel diseases. World J. Gastrointest. Pharmacol. Ther..

[41] El Sayed S., Patik I., Redhu N.S., Glickman J.N., Karagiannis K., El Naenaeey E.S.Y., Elmowalid G.A., Abd El Wahab A.M., Snapper S.B., Horwitz B.H. (2022). CCR2 promotes monocyte recruitment and intestinal inflammation in mice lacking the interleukin-10 receptor. Sci. Rep..

[42] Yang Z.J., Wang B.Y., Wang T.T., Wang F.F., Guo Y.X., Hua R.X., Shang H.W., Lu X., Xu J.D. (2021). Functions of dendritic cells and its association with intestinal diseases. Cells.

[43] Jones L.G., Vaida A., Thompson L.M., Ikuomola F.I., Caamano J.H., Burkitt M.D., Miyajima F., Williams J.M., Campbell B.J., Pritchard D.M. (2019). NF-κB2 signalling in enteroids modulates enterocyte responses to secreted factors from bone marrow-derived dendritic cells. Cell Death Dis..

[44] Shi G., Li D., Ren J., Li X., Wang T., Dou H., Hou Y. (2019). mTOR inhibitor INK128 attenuates dextran sodium sulfate-induced colitis by promotion of MDSCs on Treg cell expansion. J. Cell. Physiol..

[45] Xie Y., Zhao Y., Shi L., Li W., Chen K., Li M., Chen X., Zhang H., Li T., Matsuzawa-Ishimoto Y. (2020). Gut epithelial TSC1/mTOR controls RIPK3-dependent necroptosis in intestinal inflammation and cancer. J. Clin. Investig..

[46] Ohtani M., Hoshii T., Fujii H., Koyasu S., Hirao A., Matsuda S. (2012). Cutting edge: mTORC1 in intestinal CD11c^+^ CD11b^+^ dendritic cells regulates intestinal homeostasis by promoting IL-10 production. J. Immunol..

[47] Zhang Z., Dong L., Jia A., Chen X., Yang Q., Wang Y., Wang Y., Liu R., Cao Y., He Y. (2020). Glucocorticoids promote the onset of acute experimental colitis and cancer by upregulating mTOR signaling in intestinal epithelial cells. Cancers.

[48] Franke A., Balschun T., Karlsen T.H., Hedderich J., May S., Lu T., Schuldt D., Nikolaus S., Rosenstiel P., Krawczak M. (2008). Replication of signals from recent studies of Crohn’s disease identifies previously unknown disease loci for ulcerative colitis. Nat. Genet..

[49] Anderson C.A., Massey D.C., Barrett J.C., Prescott N.J., Tremelling M., Fisher S.A., Gwilliam R., Jacob J., Nimmo E.R., Drummond H. (2009). Investigation of Crohn’s disease risk loci in ulcerative colitis further defines their molecular relationship. Gastroenterology.

[50] Duerr R.H., Taylor K.D., Brant S.R., Rioux J.D., Silverberg M.S., Daly M.J., Steinhart A.H., Abraham C., Regueiro M., Griffiths A. (2006). A genome-wide association study identifies IL23R as an inflammatory bowel disease gene. Science.

[51] Barrett J.C., Hansoul S., Nicolae D.L., Cho J.H., Duerr R.H., Rioux J.D., Brant S.R., Silverberg M.S., Taylor K.D., Barmada M.M. (2008). Genome-wide association defines more than 30 distinct susceptibility loci for Crohn’s disease. Nat. Genet..

[52] Brand S. (2009). Crohn’s disease: Th1, Th17 or both? The change of a paradigm: New immunological and genetic insights implicate Th17 cells in the pathogenesis of Crohn’s disease. Gut.

[53] Singh B., Read S., Asseman C., Malmstrom V., Mottet C., Stephens L.A., Stepankova R., Tlaskalova H., Powrie F. (2001). Control of intestinal inflammation by regulatory T cells. Immunol. Rev..

[54] Round J.L., Mazmanian S.K. (2010). Inducible Foxp3^+^ regulatory T-cell development by a commensal bacterium of the intestinal microbiota. Proc. Natl. Acad. Sci. USA.

[55] Monteleone I., Pallone F., Monteleone G. (2011). Th17-related cytokines: New players in the control of chronic intestinal inflammation. BMC Med..

[56] O’Connor W., Kamanaka M., Booth C.J., Town T., Nakae S., Iwakura Y., Kolls J.K., Flavell R.A. (2009). A protective function for interleukin 17A in T cell-mediated intestinal inflammation. Nat. Immunol..

[57] Wedebye Schmidt E.G., Larsen H.L., Kristensen N.N., Poulsen S.S., Lynge Pedersen A.M., Claesson M.H., Pedersen A.E. (2013). TH17 cell induction and effects of IL-17A and IL-17F blockade in experimental colitis. Inflamm. Bowel Dis..

[58] Hueber W., Sands B.E., Lewitzky S., Vandemeulebroecke M., Reinisch W., Higgins P.D., Wehkamp J., Feagan B.G., Yao M.D., Karczewski M. (2012). Secukinumab, a human anti-IL-17A monoclonal antibody, for moderate to severe Crohn’s disease: Unexpected results of a randomised, double-blind placebo-controlled trial. Gut.

[59] Langley R.G., Elewski B.E., Lebwohl M., Reich K., Griffiths C.E., Papp K., Puig L., Nakagawa H., Spelman L., Sigurgeirsson B. (2014). Secukinumab in plaque psoriasis--results of two phase 3 trials. N. Engl. J. Med..

[60] Herrlinger K.R., Diculescu M., Fellermann K., Hartmann H., Howaldt S., Nikolov R., Petrov A., Reindl W., Otte J.M., Stoynov S. (2013). Efficacy, safety and tolerability of vidofludimus in patients with inflammatory bowel disease: The ENTRANCE study. J. Crohns Colitis.

[61] Uhlig H.H., Powrie F. (2018). Translating immunology into therapeutic concepts for inflammatory bowel disease. Annu. Rev. Immunol..

[62] Plichta D.R., Graham D.B., Subramanian S., Xavier R.J. (2019). Therapeutic opportunities in inflammatory bowel disease: Mechanistic dissection of host-microbiome relationships. Cell.

[63] Duan Y., Zhang E., Fang R.H., Gao W., Zhang L. (2023). Capsulated cellular nanosponges for the treatment of experimental inflammatory bowel disease. ACS Nano.

[64] Maloy K.J., Powrie F. (2011). Intestinal homeostasis and its breakdown in inflammatory bowel disease. Nature.

[65] Xu Y., Marck P., Huang M., Xie J.X., Wang T., Shapiro J.I., Cai L., Feng F., Xie Z. (2021). Biased effect of cardiotonic steroids on Na/K-ATPase-mediated signal transduction. Mol. Pharmacol..

[66] Hauptman P.J., Kelly R.A. (1999). Digitalis. Circulation.

[67] Leite J.A., Alves A.K., Galvao J.G., Teixeira M.P., Cavalcante-Silva L.H., Scavone C., Morrot A., Rumjanek V.M., Rodrigues-Mascarenhas S. (2015). Ouabain modulates zymosan-induced peritonitis in mice. Mediat. Inflamm..

[68] Galvao J., Cavalcante-Silva L.H.A., de Almeida Lima E., Carvalho D.C.M., Alves A.F., Mascarenhas S.R. (2022). Ouabain modulates airway remodeling caused by Th2-high asthma in mice. Int. Immunopharmacol..

[69] Cavalcante-Silva L.H.A., Carvalho D.C.M., de Almeida Lima E., Rodrigues-Mascarenhas S. (2021). Ouabain inhibits p38 activation in mice neutrophils. Inflammopharmacology.

[70] Carvalho D.C.M., Cavalcante-Silva L.H.A., Lima E.A., Galvao J., Alves A.K.A., Feijo P.R.O., Quintas L.E.M., Rodrigues-Mascarenhas S. (2019). Marinobufagenin inhibits neutrophil migration and proinflammatory cytokines. J. Immunol. Res..

[71] Haas M., Wang H., Tian J., Xie Z. (2002). Src-mediated inter-receptor cross-talk between the Na^+^/K^+^-ATPase and the epidermal growth factor receptor relays the signal from ouabain to mitogen-activated protein kinases. J. Biol. Chem..

[72] Fender J., Klocker J., Boivin-Jahns V., Ravens U., Jahns R., Lorenz K. (2024). “Cardiac glycosides”-quo vaditis?-past, present, and future?. Naunyn Schmiedebergs Arch. Pharmacol..

[73] Whayne T.F. (2018). Clinical use of digitalis: A state of the art review. Am. J. Cardiovasc. Drugs.

[74] Xiao S., Yosef N., Yang J., Wang Y., Zhou L., Zhu C., Wu C., Baloglu E., Schmidt D., Ramesh R. (2014). Small-molecule RORγt antagonists inhibit T helper 17 cell transcriptional network by divergent mechanisms. Immunity.

[75] Huh J.R., Leung M.W., Huang P., Ryan D.A., Krout M.R., Malapaka R.R., Chow J., Manel N., Ciofani M., Kim S.V. (2011). Digoxin and its derivatives suppress Th17 cell differentiation by antagonizing RORγt activity. Nature.

[76] Lee J., Baek S., Lee J., Lee J., Lee D.G., Park M.K., Cho M.L., Park S.H., Kwok S.K. (2015). Digoxin ameliorates autoimmune arthritis via suppression of Th17 differentiation. Int. Immunopharmacol..

[77] Tani S., Takano R., Tamura S., Oishi S., Iwaizumi M., Hamaya Y., Takagaki K., Nagata T., Seto S., Horii T. (2017). Digoxin attenuates murine experimental colitis by downregulating Th17-related cytokines. Inflamm. Bowel Dis..

[78] da Silva J.M.C., Azevedo A.D.N., Barbosa R., Teixeira M.P., Vianna T.A.G., Fittipaldi J., Cabral V.R., Paiva L.S. (2019). Ouabain decreases regulatory T cell number in mice by reducing IL-2 secretion. Neuroimmunomodulation.

[79] Jacob P.L., Leite J.A., Alves A.K., Rodrigues Y.K., Amorim F.M., Neris P.L., Oliveira M.R., Rodrigues-Mascarenhas S. (2013). Immunomodulatory activity of ouabain in *Leishmania leishmania* amazonensis-infected *Swiss* mice. Parasitol. Res..

[80] Rawat K., Shrivastava A. (2022). Neutrophils as emerging protagonists and targets in chronic inflammatory diseases. Inflamm. Res..

[81] Friedrich M., Pohin M., Powrie F. (2019). Cytokine networks in the pathophysiology of inflammatory bowel disease. Immunity.

[82] Ferreira D.A., Medeiros A.B.A., Soares M.M., Lima E.A., Oliveira G., Leite M., Machado M.V., Villar J., Barbosa L.A., Scavone C. (2024). Evaluation of anti-inflammatory activity of the new cardiotonic steroid gamma-benzylidene digoxin 8 (BD-8) in mice. Cells.

[83] Ponce A., Larre I., Castillo A., Garcia-Villegas R., Romero A., Flores-Maldonado C., Martinez-Rendon J., Contreras R.G., Cereijido M. (2014). Ouabain increases gap junctional communication in epithelial cells. Cell. Physiol. Biochem..

[84] Larre I., Lazaro A., Contreras R.G., Balda M.S., Matter K., Flores-Maldonado C., Ponce A., Flores-Benitez D., Rincon-Heredia R., Padilla-Benavides T. (2010). Ouabain modulates epithelial cell tight junction. Proc. Natl. Acad. Sci. USA.

[85] Kinoshita P.F., Yshii L.M., Vasconcelos A.R., Orellana A.M., Lima Lde S., Davel A.P., Rossoni L.V., Kawamoto E.M., Scavone C. (2014). Signaling function of Na,K-ATPase induced by ouabain against LPS as an inflammation model in hippocampus. J. Neuroinflammation.

[86] Mazala-de-Oliveira T., de Figueiredo C.S., de Rezende Correa G., da Silva M.S., Miranda R.L., de Azevedo M.A., Cossenza M., Dos Santos A.A., Giestal-de-Araujo E. (2022). Ouabain-Na^+^/K^+^-ATPase signaling regulates retinal neuroinflammation and ROS production preventing neuronal death by an autophagy-dependent mechanism following optic nerve axotomy in vitro. Neurochem. Res..

[87] Galvão J.G.F.M., Cavalcante-Silva L.H.A., Carvalho D.C.M., Ferreira L.K.D.P., Monteiro T.M., Alves A.F., Ferreira L.A.M.P., Gadelha F.A.A.F., Piuvezam M.R., Rodrigues-Mascarenhas S. (2017). Ouabain attenuates ovalbumin-induced airway inflammation. Inflamm. Res..

[88] Blaustein M.P., Hamlyn J.M. (2020). Ouabain, endogenous ouabain and ouabain-like factors: The Na^+^ pump/ouabain receptor, its linkage to NCX, and its myriad functions. Cell Calcium.

[89] Ezike T.C., Okpala U.S., Onoja U.L., Nwike C.P., Ezeako E.C., Okpara O.J., Okoroafor C.C., Eze S.C., Kalu O.L., Odoh E.C. (2023). Advances in drug delivery systems, challenges and future directions. Heliyon.

[90] Snelson M., RMuralitharan R., Liu C.F., Markó L., Forslund S.K., Marques F.Z., Tang W.W. (2025). Gut-heart axis: The role of gut microbiota and metabolites in heart failure. Circ. Res..

[91] Brennan C.A., Garrett W.S. (2016). Gut microbiota, inflammation, and colorectal cancer. Annu. Rev. Microbiol..

[92] Atarashi K., Tanoue T., Oshima K., Suda W., Nagano Y., Nishikawa H., Fukuda S., Saito T., Narushima S., Hase K. (2013). T_reg_ induction by a rationally selected mixture of Clostridia strains from the human microbiota. Nature.

[93] Atarashi K., Tanoue T., Shima T., Imaoka A., Kuwahara T., Momose Y., Cheng G., Yamasaki S., Saito T., Ohba Y. (2011). Induction of colonic regulatory T cells by indigenous *Clostridium* species. Science.

[94] Zhang X., Han Y., Huang W., Jin M., Gao Z. (2021). The influence of the gut microbiota on the bioavailability of oral drugs. Acta Pharm. Sin. B.

[95] Saha J.R., Butler V.P., Neu H.C., Lindenbaum J. (1983). Digoxin-inactivating bacteria: Identification in human gut flora. Science.

[96] Lindenbaum J., Rund D.G., Butler V.P., Tse-Eng D., Saha J.R. (1981). Inactivation of digoxin by the gut flora: Reversal by antibiotic therapy. N. Engl. J. Med..

[97] Haiser H.J., Gootenberg D.B., Chatman K., Sirasani G., Balskus E.P., Turnbaugh P.J. (2013). Predicting and manipulating cardiac drug inactivation by the human gut bacterium *Eggerthella lenta*. Science.

[98] Haiser H.J., Seim K.L., Balskus E.P., Turnbaugh P.J. (2014). Mechanistic insight into digoxin inactivation by *Eggerthella lenta* augments our understanding of its pharmacokinetics. Gut Microbes.

[99] Kumar K., Jaiswal S.K., Dhoke G.V., Srivastava G.N., Sharma A.K., Sharma V.K. (2018). Mechanistic and structural insight into promiscuity based metabolism of cardiac drug digoxin by gut microbial enzyme. J. Cell. Biochem..

[100] Sperry J.F., Wilkins T.D. (1976). Arginine, a growth-limiting factor for *Eubacterium lentum*. J. Bacteriol..

[101] Doestzada M., Vila A.V., Zhernakova A., Koonen D.P.Y., Weersma R.K., Touw D.J., Kuipers F., Wijmenga C., Fu J. (2018). Pharmacomicrobiomics: A novel route towards personalized medicine?. Protein Cell.

[102] Alexander M., Ang Q.Y., Nayak R.R., Bustion A.E., Sandy M., Zhang B., Upadhyay V., Pollard K.S., Lynch S.V., Turnbaugh P.J. (2022). Human gut bacterial metabolism drives Th17 activation and colitis. Cell Host Microbe.

